# Enhanced Wound Healing and Autogenesis Through Lentiviral Transfection of Adipose-Derived Stem Cells Combined with Dermal Substitute

**DOI:** 10.3390/biomedicines12122844

**Published:** 2024-12-13

**Authors:** Shiqi Wang, Dinghui Gao, Mingyu Li, Qian Wang, Xuanyu Du, Siming Yuan

**Affiliations:** 1 Department of Plastic Surgery, Jinling Hospital, Affiliated Hospital of Medical School, Nanjing University, Nanjing 210002, China; wangshiqi@smail.nju.edu.cn (S.W.); gaodinghui776@163.com (D.G.); limingyuu33@163.com (M.L.); 2 Department of Plastic Surgery, Jinling Hospital, Nanjing School of Clinical Medicine, Southern Medical University, Nanjing 210002, China; wang_qian2020@163.com; 3 Department of Plastic Surgery, Jinling Hospital, School of Medicine, Southeast University, Nanjing 210002, China; duxuanyumacro@foxmail.com

**Keywords:** adipose-derived stem cells (ADSCs), gene transfection, keratinocyte differentiation, dermal substitutes, wound healing

## Abstract

Background: Burns and chronic ulcers may cause severe skin loss, leading to critical health issues like shock, infection, sepsis, and multiple organ failure. Effective healing of full-thickness wounds may be challenging, with traditional methods facing limitations due to tissue shortage, infection, and lack of structural support. Methods: This study explored the combined use of gene transfection and dermal substitutes to improve wound healing. We used the DGTM (genes: *DNP63A*, *GRHL2*, *TFAP2A*, and *MYC*) factors to transfect adipose-derived stem cells (ADSCs), inducing their differentiation into keratinocytes. These transfected ADSCs were then incorporated into Pelnac^®^ dermal substitutes to enhance vascularization and cellular proliferation for better healing outcomes. Results: Gene transfer using DGTM factors successfully induced keratinocyte differentiation in ADSCs. The application of these differentiated cells with Pelnac^®^ dermal substitute to dermal wounds in mice resulted in the formation of skin tissue with a normal epidermal layer and proper collagen organization. This method alleviates the tediousness of the multiple transfection steps in previous protocols and the safety issues caused by using viral transfection reagents directly on the wound. Additionally, the inclusion of dermal substitutes addressed the lack of collagen and elastic fibers, promoting the formation of tissue resembling healthy skin rather than scar tissue. Conclusion: Integrating DGTM factor-transfected ADSCs with dermal substitutes represents a novel strategy for enhancing the healing of full-thickness wounds. Further research and clinical trials are warranted to optimize and validate this innovative approach for broader clinical applications.

## 1. Introduction

Burns, ranked as the fourth most prevalent form of trauma globally [1], and chronic ulcers, linked to a rising occurrence of daily healing [2], both may cause complete loss of skin, breakdown of the skin barrier, and significant loss of bodily fluids. They may result in shock, infection, disruptions in electrolyte levels, and imbalances in the internal environment, which can ultimately progress to sepsis, multiple organ failure, and mortality [1,3]. There is a pressing need to quickly heal full-thickness wounds. Commonly employed clinical approaches for this purpose include skin grafting [4], debridement, traditional dressings such as gauze and bandages [5,6], and bioengineered skin [7]. Nevertheless, the management of extensive chronic wounds encounters notable obstacles as a result of insufficient sources for tissue transplantation, the presence of infection, and the absence of structural reinforcement from dermal substitutes.

Adipose-derived stem cells (ADSCs) are a type of mesenchymal stem cell (MSC) that may be extracted from adipose tissue. These cells have the ability to differentiate into many cell types, tissues, and organs [8]. ADSCs are more readily available and can be cultivated more effortlessly in comparison to other MSCs. In addition, it is highly unlikely for ADSCs to elicit an immunological response [9,10]. ADSCs have demonstrated the lowest incidence of problems, including donor tissue necrosis, poor cell viability, and infection, based on clinical trials. Due to these advantages, it is currently the most widely used MSC in the field of plastic surgery [11]. In the realm of wound healing, the behavior of ADSCs can be controlled by a variety of substances that can be activated to encourage their differentiation into particular cell types. In addition, they have the ability to control the growth of new blood vessels, mobility, the formation of fibroblasts, and the recruitment of macrophages [12]. They appear to meet the requirements for an ideal cell therapy in the realm of regenerative medicine.

Dermal substitutes are polymer materials that are synthesized via biological processes and closely resemble the structure of the dermis [13,14,15,16]. They have the ability to stimulate the regeneration of the dermis. After the transplantation, fibroblasts, monocytes, and neovascularization are stimulated to slowly fill the empty space and generate extracellular matrix, which replaces the initial collagen sponges and creates a new dermal structure. Commonly used dermal regeneration substitutes include Integra^®^, which is made from bovine collagen [13]; Pelnac^®^, which is made from porcine collagen [14]; synthetic NovoSorb^®^ [15]; Matriderm^®^ [16]; and other similar products. The uppermost layer of Pelnac^®^ and Integra^®^ consists of a silicone membrane, which primarily functions to protect the wound and inhibit excessive water loss. The lowermost layer consists of a collagen sponge that promotes dermal regeneration and the formation of blood vessels [14]. Research has shown that MSCs are capable of attaching, multiplying, and moving on dermal substitutes [17].

**Figure 1:**
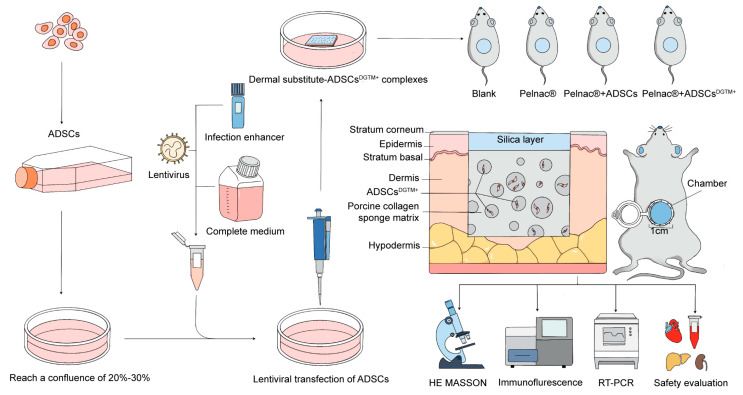
Diagrammatic summary of this study.

The gene expression patterns of keratinocytes and dermal fibroblasts have been compared by Kurita et al. They demonstrated that the transduction of the genes *DNP63A* and *GRHL2* reprogrammed ADSCs into cells resembling complex epithelial progenitors, achieved through continuous retroviral transfection. Additionally, the expression of *MYC* significantly enhanced reprogramming efficiency, cellular proliferation, and epithelial stratification, while the gene *TFAP2A* facilitated the rapid emergence of colonies. In a murine dermal wound model, the combined transduction of *DNP63A*, *GRHL2*, *TFAP2A*, and *MYC* (referred to as the DGTM factors) was shown to be sufficient to stimulate wound-site epidermal tissue growth [18]. Nevertheless, this method involves numerous in vitro transfection steps and poses safety risks due to the direct application of the viral transfection solution to the wound. Furthermore, gene transfer in isolation is insufficient to induce the formation of the dermis. In the absence of collagen and elastic fibers, the skin takes on the appearance of scar tissue rather than that of healthy skin. In our study, we combined transfection reprogramming-induced differentiation techniques with dermal substitutes. Specifically, we hypothesized that the transfection of DGTM factors into clinically harvested adipose-derived stem cells (ADSCs) would induce their differentiation into keratinocyte-like cells. Furthermore, we hypothesized that integrating these differentiated ADSCs with Pelnac^®^ dermal substitutes would enable the one-step filling of skin defects, promote enhanced angiogenesis, and stimulate cell proliferation, ultimately leading to improved wound healing. By combining genetically modified ADSCs with Pelnac^®^, our goal was to provide both cellular and structural support for the healing process, enhancing tissue regeneration while minimizing scar formation. The abstract of the article is shown in Figure 1.

## 2. Materials and Methods

### 2.1. Reagents and Antibodies

Antibodies against human CD45 (cat. no. 304012; BioLegend), CD34 (cat. no. 343509; BioLegend), CD105 (cat. no. 323205; BioLegend), CD73 (Ecto-5′-nucleotidase) (cat. no. 344003; BioLegend), and CD90 (Thy1) (cat. no. 328109; BioLegend) were obtained from BioLegend (San Diego, CA, USA). HiScript II Q RT SuperMix for qPCR (cat. no. R222-01; Vazyme) and ChamQ Universal SYBR qPCR Master Mix (cat. no. Q711-02; Vazyme) were obtained from Vazyme (Beijing, China). Human MSC osteogenic differentiation kit (cat. no. PWL080, Meilunbio) and Human MSC adipogenic differentiation kit (cat. no. PWL081, Meilunbio) was obtained from Meilunbio (Nanjing, China). E-Cadherin (24E10) rabbit mAb (cat. no. 3195S; Danvers, MA, USA), Anti-Cytokeratin 14 antibody [LL002] (cat. no. ab7800-100 ug; Abcam, Cambridge, UK), goat anti-mouse IgG-HRP (cat. no. abs20039ss; Absin, Shanghai, China), mouse-derived GAPDH (cat. no. RF230546A; Thermo Fisher Scientific, Waltham, MA, USA), rabbit-derived GAPDH (cat. no. AB-P-R-001; Good Here Biotechnology, Hangzhou, China), 0.1 M sodium dimethylarsenate buffer (Sigma-Aldrich, St. Louis, MO, USA) and RIPA protein lysis buffer (Santa Cruz Biotechnology, Santa Cruz, CA, USA) were obtained from the respective manufacturers. GAPDH was used as a protein loading control. Lentivirus transfection enhancers HiTransG A and HiTransG P were obtained from GeneChem Co., Ltd. (Shanghai, China). The complete medium for human epidermal keratinocytes (KSF) (cat. no. PCM-H-090; Zhongqiao, Xinzhou, China) was purchased from Shanghai Zhongqiao Xinzhou Biotechnology Co., Ltd. (Shanghai, China). The Pelnac^®^ was purchased from Gunze Corporation (Kyoto, Japan). Dulbecco’s Modified Eagle Medium (DMEM), Fetal Bovine Serum (FBS), and Phosphate-Buffered Saline (PBS) were purchased from Gibco (Grand Island, NY, USA).

### 2.2. Isolation, Culture, and Identification of ADSCs

Subcutaneous adipose tissue from patients undergoing abdominal surgery was collected under aseptic conditions. Soft tissues and small blood vessels were removed as much as possible, rinsed repeatedly with PBS to remove blood cells, and then digested with 0.1% type I collagenase. The effect of collagenase was terminated by the neutralization of DMEM with 20% FBS, and then the extracellular matrix was removed by centrifugation for 10 min at 3000 rpm. The cells were then transferred into centrifugation tubes and resuspended in DMEM. The cells were then centrifuged at 3000 rpm for 10 min, after which they were resuspended in DMEM with 20% FBS. They were then inoculated into Petri dishes and cultured in an incubator at 37 °C with 5% CO_2_ for 48 h. After 48 h, the non-adherent cells were removed, and the liquid was replaced with a new one for the following three days. The growth of the cells was observed and photographed under a phase contrast microscope on a daily basis. It was observed that the adhered cells had grown to occupy the majority of the bottom wall of the Petri dishes (80% to 90%) after 10 to 14 days of culture. The percentage of adhered cells was found to be 90%. To characterize the phenotype of ADSCs, the expression of cell surface markers CD90, CD105, CD73, CD45, and CD34 was quantified by flow cytometry. ADSCs were cultured in the human adipose MSC-induced differentiation kit to induce osteogenic and lipogenic differentiation. The differentiation of osteoblasts or adipocytes was observed through the use of alizarin red or oil red O staining, respectively. This study was reviewed by the Ethics Committee of the Eastern Theater General Hospital (No. 2024DZSJJ-020).

### 2.3. Lentiviral Transfection of ADSCs to Characterize Transfection Efficiency and Cellular Properties

The lentivirus was prepared by Shanghai GeneChem Co., Ltd., and details regarding the viral gene information and vector can be found in the Supplementary Table S1. Due to the need to transfect four genes using two lentiviruses, and in the absence of infection enhancers, our experiments revealed that the transfection efficiency was relatively low. To improve this, we employed two self-developed infection enhancers from GeneChem Co., Ltd. (Shanghai, China): HiTransG A and HiTransG P. HiTransG A is a large non-ionic amphiphilic molecule that significantly enhances lentiviral infection efficiency without inducing cytotoxicity. HiTransG P is a cationic polymer that boosts lentiviral infection by reducing the charge repulsion between the cell membrane and the virus. Notably, both enhancers are considerably less cytotoxic than Polybrene [19]. ADSCs were seeded in 96-well plates at a density of 3 × 10^3^ cells per well, with a total of 12 wells. Three wells were designated as group M and maintained in a complete medium to observe the infection effect of the virus on the cells under conventional culture conditions. Three wells were designated as group A, utilizing a complete medium with 4 µL viral enhancement solution HiTransG A, to assess the potential of HiTransG A to augment the infection efficacy. Similarly, three wells were designated as group P, employing a complete medium with 4 µL HiTransG P, to evaluate the ability of HiTransG P to enhance the infection efficacy. The remaining three wells were designated as the control group, whose purpose was to monitor the normal growth of cells during the experiment. The cells were incubated at 37 °C for 24 h until they reached a confluence of 20–30%. The virus should be removed from the refrigerator and melted slowly on ice. The virus should be diluted in complete medium to a titer of 1 × 10^8^ TU/mL, 5 × 10^7^ TU/mL, or 1 × 10^7^ TU/mL, with at least 35 µL in each group after completion of the dilution. The supernatant was aspirated from each well, the virus and the appropriate infection enhancer were added, and the mixture was mixed before continuing the incubation. At 16 h post-infection, the medium was changed to KSF medium. During this process, cell morphology was observed. If necessary, the medium could be changed 8 h earlier to maintain the normal growth of the cells when changes occurred. The culture continued until 72 h had elapsed, at which point the fluid was changed as necessary. The cells were observed under a microscope when the fluorescence expression was abundant. The infection conditions and MOI corresponding to the group with approximately 80% infection efficiency and good cell growth were then used as the basis for subsequent infection experiments. Puromycin and neomycin were employed for cell screening following successful transfection, and only the cells that tested positive for transfection were retained and cultured on the KSF professional medium for subsequent identification and further investigation. The expression status of the transfected gene in ADSCs was determined using RT-PCR. Transfected cells were analyzed using a Western blot assay to detect the presence of the keratinocyte-specific markers Keratin 14 (KRT14) and Cadherin 1 (CDH1). The presence of the keratinocyte-specific marker KRT14 was identified in the transfected cells by applying immunohistochemistry and immunofluorescence techniques.

### 2.4. Construction of Dermal Substitute–ADSCs^DGTM+^ Complexes

In this study, we used Pelnac^®^ as a dermal substitute in our experimental procedures. Pelnac^®^ was chosen because it is commonly used in the clinics of the hospitals affiliated with our laboratory, which facilitates its accessibility for our research. We declare that there is no commercial interest involved. Transfected ADSCs were seeded onto the dermal substitute at a density of 3 × 10^4^/mL. Three days after the inoculation of cells on dermal substitutes, the cultures were fixed in 4% paraformaldehyde for 30 min, stained with DAPI, and observed under a confocal microscope (Leica DMI6000, Leica Microsystems GmbH, Wetzlar, Germany).

Forty-eight hours after inoculation of cells on dermal substitutes, cultures were fixed in a solution of 2.5% glutaraldehyde in 0.1 M sodium dimethylarsenate buffer for twelve hours at 4 °C. They were then washed in sodium dimethylarsenate buffer and post-fixed in a solution of 1% osmium tetroxide for 2 h. Following dehydration (30%, 50%, 70%, 90%, and 100% ethanol), the cultures were dried in a critical point CO₂ apparatus (Leica MS CPD 030, Leica Microsystems GmbH, Wetzlar, Germany) and metalized with a 30 nm gold overlay (Leica EM SCD 500, Leica Microsystems GmbH, Wetzlar, Germany), and electrons were captured in a scanning electron microscope (Jeol JSM-6390LV, EOL Ltd., Akishima, Tokyo, Japan) by side illumination at 15 kV for analysis.

### 2.5. Animal Experimentation

Four groups were established: blank group, Pelnac^®^ group (only dermal substitute was placed), ADSCs + Pelnac^®^ group (wild-type cells combined with Pelnac^®^ group) and ADSCs^DTGM+^ + Pelnac^®^ group. Each group consisted of 4 C57BL/6J male mice, aged 6–8 weeks, purchased from Jicui Yaka BioTech Co., Ltd. (Nanjing, China), and housed in an SPF-grade animal facility. The wound model was established based on previous studies [18] as follows: A chamber was created by cutting a 1.5 mL Eppendorf tube. The cut was then heated with a flame to smooth and widen it. Two layers of holes were made using a 22-gauge needle, which was also heated with a flame. After tanning the inner surface with an electric file, the chamber was autoclaved. Under isoflurane general anesthesia, the chamber connections were shaved and sterilized. Subsequently, a circle of skin and subcutaneous tissue with a diameter of 1 cm was excised subcutaneously, and the chambers were sutured to the deep fascia with 4-0 horizontal mattress sutures. On the other hand, cells successfully transfected with DGTM factor after screening with puromycin and neomycin as previously described were digested and resuspended into a cell suspension. A total of 200 μL of cell suspension with a density of 2 × 10^5^/mL was seeded into the dermal layer. The wound was covered with a dermal substitute collagen sponge facing downwards and secured with sutures and fixators. The procedure was completed with appropriate pressure and wrapping. In order to assess the regeneration of epithelial tissue from the base of the skin ulcer, photographs were taken every two days under inhalation anesthesia. The Pelnac^®^ silicone membrane was removed at three days postoperatively, and the Eppendorf tube lumen was cut one week postoperatively. Mice were executed on the 13th day after surgery, and skin specimens were taken. HE staining was employed to observe the regenerative dermatization of the new skin, while MASSON staining was utilized to compare the arrangement of fibers in the dermis. Immunofluorescence anti-Ki67 and anti-CD31 were utilized to observe cell proliferation and neovascularization. After the treatment, peripheral blood samples were collected from the mice to assess the presence of lentivirus, perform routine blood tests, analyze blood biochemistry, and evaluate immune-related indices for any abnormalities. Additionally, liver and kidney samples were obtained for histopathological examination to assess potential damage. The animal experiments conducted in this study were reviewed and approved by the Committee for the Use and Management of Laboratory Animals of the Jinling Hospital (No. DZISSDWLS240024).

### 2.6. RT-qPCR

To extract total RNA, cells were collected and lysed with 1 mL of TRIzol reagent, followed by the addition of 200 μL of chloroform. After centrifugation (12,000× *g*, 15 min, 4 °C), total RNA in the supernatant was precipitated with isopropanol. The RNA pellet was washed with 75% ethanol at 4 °C, and its purity and concentration were measured by OD reading. cDNA was synthesized using HiScript II Q RT SuperMix for qPCR in a PCR instrument (T100™ Thermal Cycler, Bio-Rad Laboratories, Inc., Hercules, CA, USA.). RT-qPCR was performed on the LineGene9600 (Hangzhou Bioer Technology Co., Ltd., Hangzhou, Zhejiang, China.) using ChamQ Universal SYBR qPCR Master Mix. Gene expression levels were normalized to the expression of GAPDH. The primers used in this study are shown in the Supplementary Table S2.

### 2.7. Western Blot

RIPA protein lysis buffer (Santa Cruz, CA, USA) containing 1% protease and phosphatase inhibitors was added to the collected cells, which were then lysed for 30 min on a shaker at 4 °C. Following lysis, the samples were centrifuged at 12,000× *g* for 15 min at 4 °C. To the resulting protein extract, 1/5 volume of 6× Protein Loading Buffer was added, and the samples were heated at 99 °C for 10 min in a metal bath. Total proteins were separated by SDS-PAGE and transferred to a polyvinylidene fluoride (PVDF) membrane (Millipore Co., Burlington, MA, USA). The membrane was blocked at room temperature for 2 h with 5% BSA in TBST (50 mM Tris/HCl, pH 7.6, 150 mM NaCl, and 0.1% Tween-20). It was then incubated overnight at 4 °C with the primary antibody, followed by three washes with TBST. The membrane was subsequently incubated for 2 h at room temperature with the appropriate HRP-conjugated secondary antibody and washed three times. Immunoreactive bands were detected using enhanced chemiluminescence (ECL) and a minichem™ chemiluminescence imaging system (SAGECREATION, Zhejiang, China). Grayscale values of the bands were analyzed using ImageJ software (version 1.52i; National Institutes of Health, Bethesda, MD, USA).

### 2.8. Statistical Analysis

Data from at least three independent studies are presented as means ± standard error. Statistical analysis for each group was performed using ANOVA with GraphPad Prism 9.5 software. For non-normally distributed data, the Mann–Whitney test and the Kruskal–Wallis test were employed. The data were deemed statistically significant when the probability (*p*) value was less than 0.05 (* *p* < 0.05), less than 0.01 (** *p* < 0.01), or less than 0.001 (*** *p* < 0.001) in comparison to the displayed groups.

## 3. Results

### 3.1. ADSCs Displaying Characteristic Surface Markers and Differentiation Potential Were Isolated and Cultured

**Figure 2:**
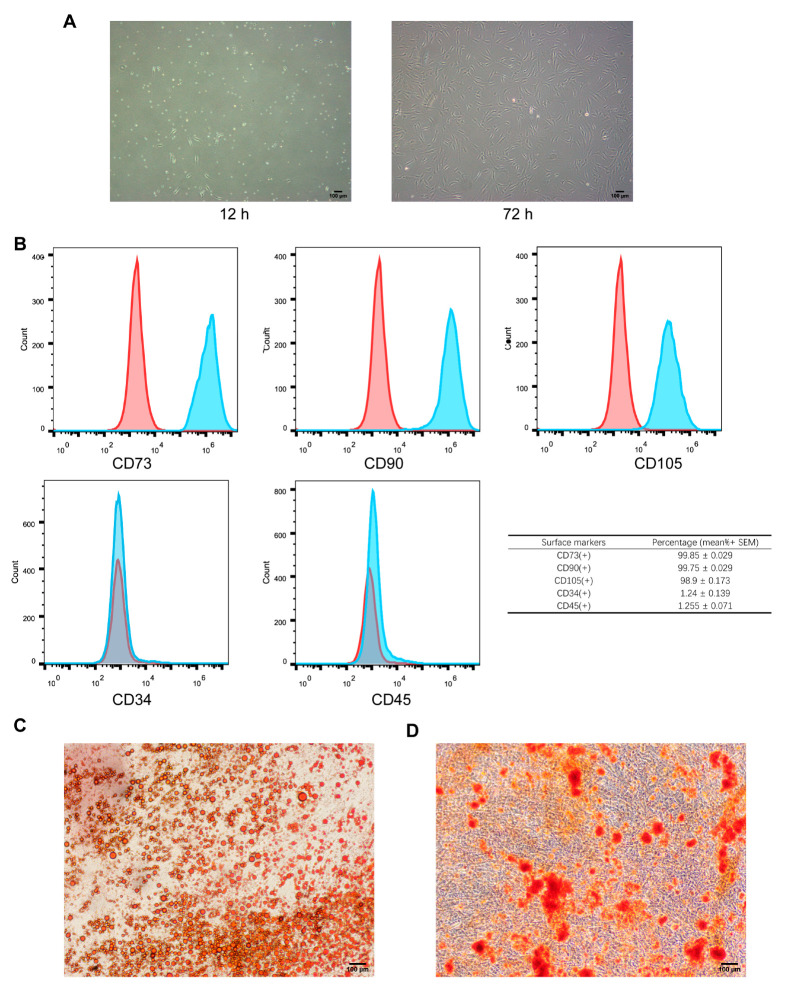
Identification of ADSCs. (**A**) A Morphology of human ADSCs at 12 h and 72 h; scale bar: 100 μm. (**B**) Flow cytometry detection of ADSCs. ADSCs were positive for the markers CD73, CD90, and CD105 (blue peaks) and negative for the markers CD34 and CD45 (red peaks). The table summarizes the percentage of cells expressing each marker (mean ± SEM). (**C**) ADSC lipogenicity assay; scale bar: 100 μm. (**D**) ADSC osteogenicity assay; scale bar: 100 μm.

After 12 h of primary cell culture, the cells displayed a compact spindle shape when observed under a microscope. They also showed clustered growth and a limited level of attachment. Following a period of 72 h, there was a noticeable increase in the volume of ADSCs. Additionally, the local ADSCs displayed shuttle, cluster, and helical growth patterns, along with a rapid rate of proliferation (Figure 2A). The flow cytometry technique was used to measure the expression of surface markers of ADSCs. The expression levels of CD90, CD73, and CD105 were greater than 98%, while the expression levels of CD34 and CD45 were less than 2%. Following the induction of lipid differentiation, the lipid droplets were dyed red using oil red O staining (Figure 2C). Following the induction of osteogenic differentiation, the cells that underwent this process exhibited the presence of calcium deposits, as evidenced by alizarin red staining. The cells that were different from each other showed an orange-red color, as depicted in Figure 2D. These results satisfy the criterion for identifying ADSCs [20].

### 3.2. Efficient Lentiviral Transfection of ADSCs^DGTM+^ and Validation of Keratinocyte-Specific Gene Expression

**Figure 3:**
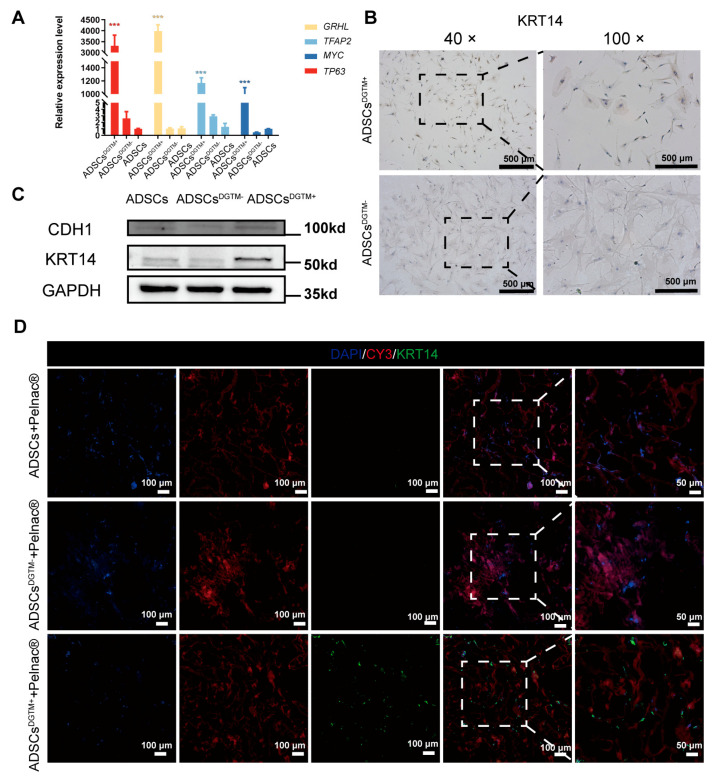
Identification of ADSCs^DGTM+^. (**A**) Statistical graph of RT-PCR detection of the expression of transfected genes *GRHL*, *TFAP2*, *MYC*, and *TP63* in ADSCs^DGTM+^, ***, *p* < 0.001. (**B**) Representative graph of immunohistochemistry detection of the expression of the keratinocyte-specific marker KRT14 in ADSCs^DGTM+^ and ADSCs^DGTM−^. (**C**) WB detection of keratinocyte-specific markers KRT14 and CDH1 expression in ADSC, ADSCs^DGTM+^ and ADSCs^DGTM-^. (**D**) Representative fluorescent staining of expression of keratinocyte-specific markers KRT14, CY3 (Pelnac^®^ staining), and DAPI in the ADSCs + Pelnac^®^ group, ADSCs^DGTM+^ + Pelnac^®^ group and ADSCs^DGTM-^ + Pelnac^®^ group.

When observed under a fluorescence microscope, cells that have undergone transfection exhibit visible green fluorescence from GFP. At a multiplicity of infection (MOI) of 10, the fluorescence ratio, when used in conjunction with the viral enhancement solution HiTransG P, was found to be between 80% and 90% (Figure S1A). Furthermore, when ADSCs were genetically modified using CCK8 with an MOI of 10 and different concentrations of the viral enhancement solution HiTransG P, it was observed that the concentration of HiTransG P at 25× showed toxicity, whereas concentrations of HiTransG P ranging from 1× to 15× did not display any noticeable harmful effects (Figure S1B–D). The optimal conditions for lentivirus transfection were found to be a HiTransG P concentration of 5× and a MOI of 10. RT-PCR was employed to detect and analyze the genes present in the transfected ADSCs (ADSCs^DGTM+^). The results, depicted in Figure 3A, demonstrated a significant positive presence of all four targeted genes. We employed immunohistochemistry to label anti-KRT14, a protein that is exclusively expressed in keratinocytes, on ADSCs^DGTM+^. The results were positive, indicating the presence of the transfected ADSCs and confirming their differentiation into keratinocytes (Figure 3B). Figure 3C,D exhibit the occurrence of the KRT14 protein and CDH1 protein, specifically expressed in keratinocytes, yielding beneficial outcomes. The data described above demonstrate that the cells were successfully transfected and able to produce the keratinocyte-specific expression protein.

### 3.3. ADSCs^DGTM+^ Exhibit Successful Adhesion, Proliferation, and Keratinocyte Differentiation on Pelnac^®^ Dermal Substitutes

**Figure 4:**
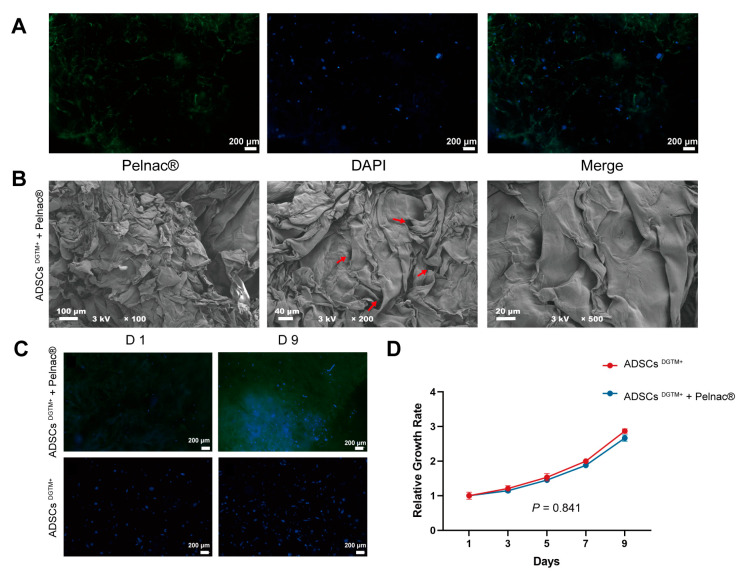
Construction of dermal substitute–ADSCs^DGTM+^ complexes. (**A**) Observation of dermal substitute–ADSCs^DGTM+^ complexes under inverted fluorescence microscope; scale bar: 200 μm. (**B**) Observation of dermal substitute–ADSCs^DGTM+^ complexes under scanning electron microscope at different magnifications. The red arrow in the middle image points to the pore. (**C**,**D**) Observation of the proliferation of ADSCs^DGTM+^ grown in Pelnac^®^ by culturing ADSCs^DGTM+^ alone within 9 days under an inverted fluorescence microscope and statistical graphs; scale bar: 200 μm.

Observations made using laser confocal microscopy (Figure 4A) indicated that the Pelnac^®^ material had an excitation light wavelength of approximately 550 nm (Figure 4A, left green). Additionally, the images showed that the ADSCs^DGTM+^ were evenly distributed and thriving on the dermal substitute Pelnac^®^. Scanning electron microscopy (SEM) was used to examine the structure and porosity of Pelnac^®^, as shown in Figure 4B, as well as the inner layer. The SEM analysis showed that ADSCs^DGTM+^ adhered to and spread effectively on the dermal matrices. CCK8 was conducted to assess the beneficial impact of the dermal substitute Pelnac^®^ on the survival and growth of ADSCs^DGTM+^. Over the course of a 9-day incubation period, the ADSCs^DGTM+^ showed a notable increase in their ability to survive and multiply on Pelnac^®^. This increase was comparable to the growth of cells cultured under normal conditions (*p* > 0.05) (Figure 4C,D). The results demonstrated that the dermal substitute positively influenced the attachment, expansion, and proliferation of ADSCs^DGTM+^ in a laboratory setting. Furthermore, the anti-KRT14 immunofluorescence assay conducted on ADSCs^DGTM+^ grown on Pelnac^®^ demonstrated positive outcomes. This suggests that the ADSCs^DGTM+^ maintained their properties after being transfected and were able to effectively produce proteins specific to keratinocytes.

### 3.4. Dermal Substitute–ADSCs^DGTM+^ Complexes Prove to Be a Highly Efficient Approach for the Restoration of Complete Skin Defect Wounds in Mice

#### 3.4.1. The Entire Skin Defect Wound Can Be Filled with Dermal Substitute–ADSCs^DGTM+^ Complexes

**Figure 5:**
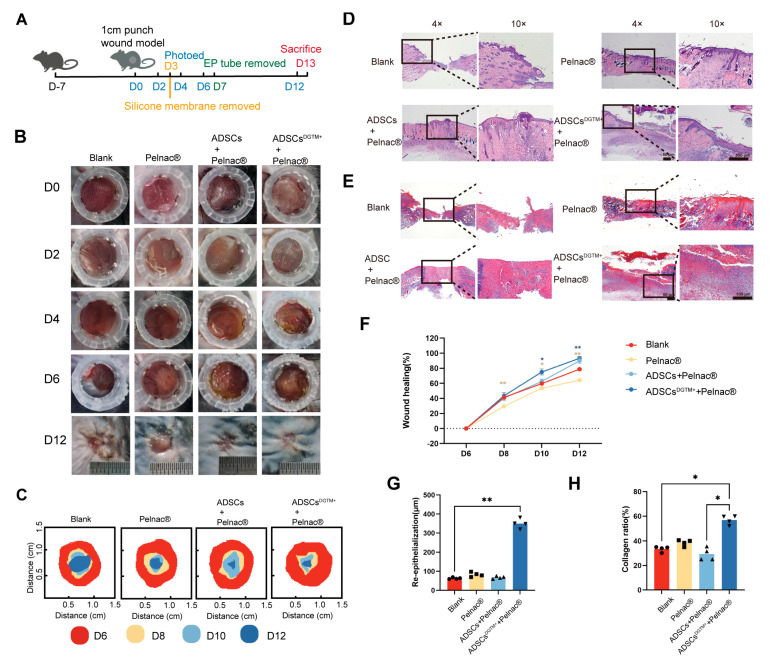
Dermal substitute–ADSCs^DGTM+^ complexes fill full-thickness defective wounds. (**A**) Flowchart of animal experiments. (**B**) Representative images of skin regeneration. Mice wounds were divided into the following groups: blank, Pelnac^®^ ADSCs + Pelnac^®^, and ADSCs^DGTM+^ + Pelnac^®^. (**C**) Temporal variation of skin regeneration. (**D**) HE staining of mice in each group of the wound. (**E**) MASSON staining of mice in each group of the wound. (**F**) Statistical graph of the percentage of wound healing over time for each group. (**G**) Statistical graph of the thickness of re-epithelialization of the wound in each group. (**H**) Statistical graph of the proportion of wound collagen in each group. * *p* < 0.05, ** *p* < 0.01, n = 4.

The ADSCs^DGTM+^ + Pelnac^®^ group showed a morphology similar to that of normal skin attached to the surface on the sixth day (Figure 5B). We sampled incompletely healed skin specimens on day 13. Compared to the blank control group, the wound healing rate was significantly higher in the ADSCs^DGTM+^ + Pelnac^®^ group on days 10 and 12 (Figure 5C,F). HE staining showed that the wounds in the Pelnac^®^-treated group had a smooth, flat filling (Figure 5D). The thickness of the newly formed epidermis in the ADSCs^DGTM+^ + Pelnac^®^ group was significantly greater than that in the blank group (*p* < 0.01, Figure 5D,G). Masson staining revealed that the collagen fibers in the blank group were more disorganized, while those in the other groups exhibited a more organized arrangement (Figure 5E). The proportion of well-organized collagen fibers was significantly higher in the ADSCs^DGTM+^ + Pelnac^®^ group compared to the blank group (*p* < 0.05, Figure 5E,H).

#### 3.4.2. Dermal Substitute–ADSCs^DGTM+^ Complex Promotes Angiogenesis and Cell Proliferation in Whole Skin Wounds

**Figure 6:**
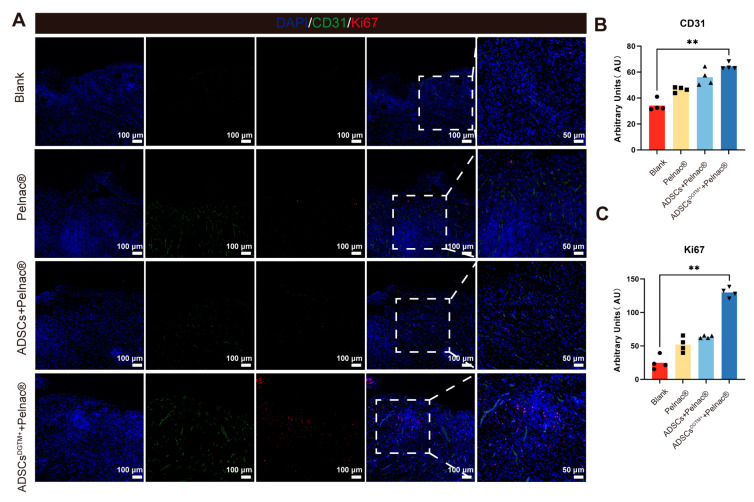
Dermal substitute–ADSCs^DGTM+^ complexes promote wound cell proliferation and revascularization. (**A**) Representative immunofluorescence staining of CD31, Ki67, and DAPI in the whole layer defect wounds of neoplastic skin of each wound group. (**B**) Immunofluorescence staining of CD31 in neoplastic whole skin defect wounds in each group. (**C**) Immunofluorescence staining of Ki67 in neoplastic whole skin defect wounds in each group; ** *p* < 0.01, n = 4.

Immunofluorescence staining with anti-Ki67 and anti-CD31 was performed on the skin of four groups of mice to label proliferating cells and neovascularization, respectively. The results demonstrated that the ADSCs^DTGM+^ + Pelnac^®^ group exhibited a higher number of Ki67^+^ cells, a marker of cell proliferation, compared to the other groups (*p* < 0.01, vs. the blank group). Additionally, the wound of the ADSCs^DTGM+^ + Pelnac^®^ group exhibited a higher expression of anti-CD31 cells (*p* < 0.01, vs. the blank group) (Figure 6). The results indicate that the application of dermal substitute–ADSCs^DGTM+^ supported the wound with skin regeneration and vascularization.

#### 3.4.3. ADSCs^DGTM+^ in the Dermal Substitute–ADSCs^DGTM+^ Complex Have the Capacity to Transform into Keratin-Forming Cells, Which Are Involved in the Filling of Full-Thickness Skin Wounds

**Figure 7:**
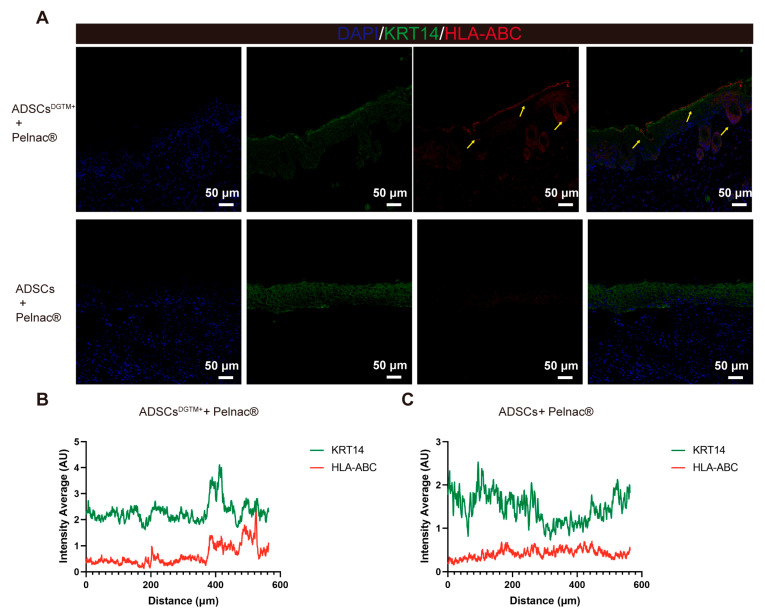
Dermal substitute–ADSCs^DGTM+^ complexes involved in the filling of full-thickness defective skin. (**A**) Representative fluorescent staining of KRT14, HLA-ABC, and DAPI in the wounds of mice in the ADSCs + Pelnac^®^ and ADSCs^DGTM+^ + Pelnac^®^ groups. (**B**,**C**) Representative plots of average fluorescence intensity of different channels in the ADSCs^DGTM+^ + Pelnac^®^ group and ADSCs + Pelnac^®^ group.

To ascertain whether the transfected ADSCs^DGTM+^ were involved in the filling of whole skin wounds, we co-stained the ADSCs + Pelnac^®^ group and ADSCs^DTGM+^ + Pelnac^®^ group wounds with the human-specific antigens HLA-ABC [21,22] and the keratinocyte-specific antigen KRT14 [18], respectively. The results demonstrated that the epidermal layer and the basal layer of hair follicles in the wound of the ADSCs^DTGM+^ + Pelnac^®^ group exhibited co-expression of HLA-ABC and KRT14, indicating that ADSCs^DGTM+^ may undergo transformation into keratin-forming cells involved in the restoration of the entire layer of wound skin (Figure 7A, yellow arrows). Fluorescence expression analysis of sections for both antigens revealed that the ADSCs^DTGM+^ + Pelnac^®^ group exhibited co-expression (Figure 7B), whereas the ADSCs + Pelnac^®^ group did not (Figure 7C). This further corroborates our conclusion.

### 3.5. Dermal Substitute–ADSCs^DGTM+^ Complex Demonstrates No Detectable Toxicity or Organ Impairment in Mice

The RT-PCR study of the peripheral blood of mice in the ADSCs^DTGM+^ + Pelnac^®^ group for repairing full-thickness skin defects showed no expression of the transcribed genes 13 days after the therapy (Figure S2B). Histological analysis of the liver and kidney, which are the principal organs for drug excretion, using HE stains, showed no discernible differences between the treated group and the control group. This suggests that the therapy did not cause any harm to these organs (Figure S2C,D). The blood samples were subjected to biochemical examination, which indicated that the glucose levels fell within the normal range for all groups. In addition, the cardiac index, creatine kinase (CK), hepatic indices aspartate aminotransferase (AST) and alanine aminotransferase (ALT), and renal indices blood urea nitrogen (BUN) and creatinine (CREA) were all within the normal range, as shown in Figure S2A. The results suggest that the dermal substitute–ADSCs^DGTM+^ therapy does not cause any harm to the heart, liver, and kidneys of mice.

## 4. Discussion

In the context of extensive skin defects resulting from burns and chronic wounds, where dermal donor sites are severely limited, it is of paramount importance to develop innovative methods for skin repair in order to restore the skin barrier in a prompt and efficacious manner. This study references and builds on the transfection methodology of a previous study [18] and further combines ADSCs that have been transfected with lentiviral DGTM factors with the dermal substitute Pelnac^®^. The combination was then tested on full-thickness skin defects in mice, resulting in enhanced cell proliferation and neovascularization, thereby promoting wound healing.

Our results demonstrate that ADSCs exhibited normal growth and proliferation in Pelnac^®^ during the nine-day co-culture period, which is consistent with findings from other studies [23,24]. Pelnac^®^ is a bilayer membrane composed of an outer silica layer and a porcine collagen sponge matrix, with pore sizes ranging from 60 to 110 µm. The inner layer supports the vascularization and colonization of dermal fibroblasts [25]. Our observations suggest that ADSCs can effectively penetrate, distribute, and proliferate within the 3D structures formed by Pelnac^®^. Previous studies have shown that the absence of dorsal receptor anchoring in 2D cultures disrupts the balance between spreading and retraction, thereby fostering an environment that stimulates the organization of plate-like pseudopods, stress fibers, and adhesion patches. In contrast, 3D culture systems alleviate the geometrical and mechanical constraints imposed by 2D environments, allowing for a more balanced distribution of growth. Moreover, 3D culture restores cell polarity, which is crucial for intracellular signaling and significantly influences cell behavior, including growth, migration, survival, and differentiation [26,27]. Dermal substitutes have been shown to induce the formation of dermal-like granulation tissue, thereby enhancing wound healing quality [28]. Based on prior studies comparing different dermal substitutes [29,30,31], it is reasonable to hypothesize that dermal substitutes like Integra^®^ could serve a similar role as Pelnac^®^—the scaffold used in our experiments. Our findings further confirm the feasibility and efficacy of combining mesenchymal stem cells (MSCs) with dermal substitutes for potential clinical applications.

The pro-healing effects of ADSCs on wounds have been documented in numerous studies. The paracrine function of ADSCs enhances angiogenesis and generates growth factors that promote neointima formation [8,10,12]. In the inflammatory wound environment, ADSCs secrete TGF-β, which, in combination with IL-1β and IL-6, promotes macrophage recruitment and facilitates the polarization of macrophages from the M1 to the M2 phenotype [32]. Additionally, ADSCs may be stimulated by IL-6 to self-induce and secrete a variety of factors, including TNF-α, b-FGF, VEGF, TLR2, TLR4, IL-6, TGF-β, and GDF11 [33]. Furthermore, extracellular vesicles derived from ADSCs play a critical role in the migration and proliferation of dermal fibroblasts and keratinocytes, as well as in collagen and elastin deposition [34,35]. ADSCs also express HIF-1α, which regulates VEGF gene expression in endothelial cells [36,37].The results of our animal experiments corroborate these findings, demonstrating an increase in the number of Ki67^+^ proliferating cells and anti-CD31 expression in all groups treated with ADSCs (compared to the blank group). These results are consistent with those of previous studies, including those by Meruane et al., who reported increased microvessel density and collagen I synthesis with ADSC-integrated dermal substitutes (Integra^®^) compared to the substitute-only group [38].

The primary objective of our study is to demonstrate that ADSCs can be utilized for the reconstruction of full-thickness skin defects. Previous research has shown that transfection of ADSCs with the DGTM factor can effectively convert them into keratinocytes, which in turn can facilitate the formation of epidermis in situ [18]. Our study replicated and streamlined this protocol and combined the transfected ADSCs^DGTM+^ with dermal substitutes in an attempt to achieve greater gains on a larger scale in patients with total skin defects. The results demonstrated the appearance of human-derived ADSCs in the epidermal and follicular layers of wound of mouse, as evidenced by HLA-ABC and KRT14 co-expression (Figure 7). Furthermore, wound closure and collagen alignment were also promoted. The low immunogenicity of ADSCs allows for the performance of this animal experiment without the induction of an immune response [9,10], thereby ensuring the safety of the procedure for use in clinical trials. In addition, not directly applying the lentiviral transfection solution to the skin also ensures the biological safety of the experiment (Figure S2).

One limitation of this study is that the simultaneous labeling of fluorescence became technically challenging due to the transfection of the four genes together. To address this challenge, we ultimately employed HLA-ABC to label ADSCs on mouse skin and utilized co-expression to identify ADSCs^DGTM+^. Further in vivo studies are needed to evaluate the long-term effects of regimens combining ADSCs with dermal substitutes, using larger animal models such as rats, rabbits, and pigs, with either larger wound areas or extended EP tube placement. These studies are crucial before advancing these approaches to clinical trials.

## 5. Conclusions

This study demonstrates the construction of a novel tissue-engineered skin. The combination of ADSCs transfected with lentiviral DGTM and a dermal substitute (Pelnac^®^) resulted in the one-step repair of full-thickness wounds with minimal skin shrinkage, increased neovascularization and proliferative cells, and a morphology close to normal skin. This approach offers a promising regenerative strategy for skin engineering, stem cell delivery, and regeneration of damaged skin tissues. It also provides a novel concept for clinical one-step composite skin implantation.

## Data Availability

The raw data supporting the conclusions of this article will be made available by the authors, without undue reservation.

## Supplementary Materials

The following supporting information can be downloaded at https://www.mdpi.com/article/10.3390/biomedicines12122844/s1: Figure S1: Lentiviral transfection of ADSCs; Figure S2: Safety evaluation. Table S1: List of plasmid vectors; Table S2: Primer sequences.
