# Supplementary material for: Enhanced Wound Healing and Autogenesis Through Lentiviral Transfection of Adipose-Derived Stem Cells Combined with Dermal Substitute
Source: Biomedicines. 2024 Dec 13;12(12):2844. doi: 10.3390/biomedicines12122844 (PMC11673073; doi:10.3390/biomedicines12122844)
Supplement: Supplementary file 1 [file biomedicines-12-02844-s001.zip › TableS2.pdf]

Table S2. Primer Sequences

Species	Gene Symbol	Primer	Sequence (5′ — 3′)
Homo sapiens	*GAPDH*	FP	GCATGGCCTTCCGTGTTCC
	GAPDH	RP	GGGTGGTCCAGGGTTTCTTACTC
	MYC	FP	GGCTCCTGGCAAAAGGTCA
	MYC	RP	CTGCGTAGTTGTGCTGATGT
	TFAP2A	FP	AGGTCAATCTCCCTACACGAG
	TFAP2A	RP	GGAGTAAGGATCTTGCGACTGG
	GRHL2	FP	GAAAACCGAGTGCAAGTCCTA
	GRHL2	RP	GGGCCATGAAAACTGGTGTG
	TP63	FP	GGACCAGCAGATTCAGAACGG
	TP63	RP	AGGACACGTCGAAACTGTGC
	KRT14	FP	TGAGCCGCATTCTGAACGAG
	KRT14	RP	GATGACTGCGATCCAGAGGA
	CDH1	FP	ATTTTTCCCTCGACACCCGAT
	CDH1	RP	TCCCAGGCGTAGACCAAGA
