# Supplementary figures and images for: Enhanced Wound Healing and Autogenesis Through Lentiviral Transfection of Adipose-Derived Stem Cells Combined with Dermal Substitute
Source: Biomedicines. 2024 Dec 13;12(12):2844. doi: 10.3390/biomedicines12122844 (PMC11673073; doi:10.3390/biomedicines12122844)

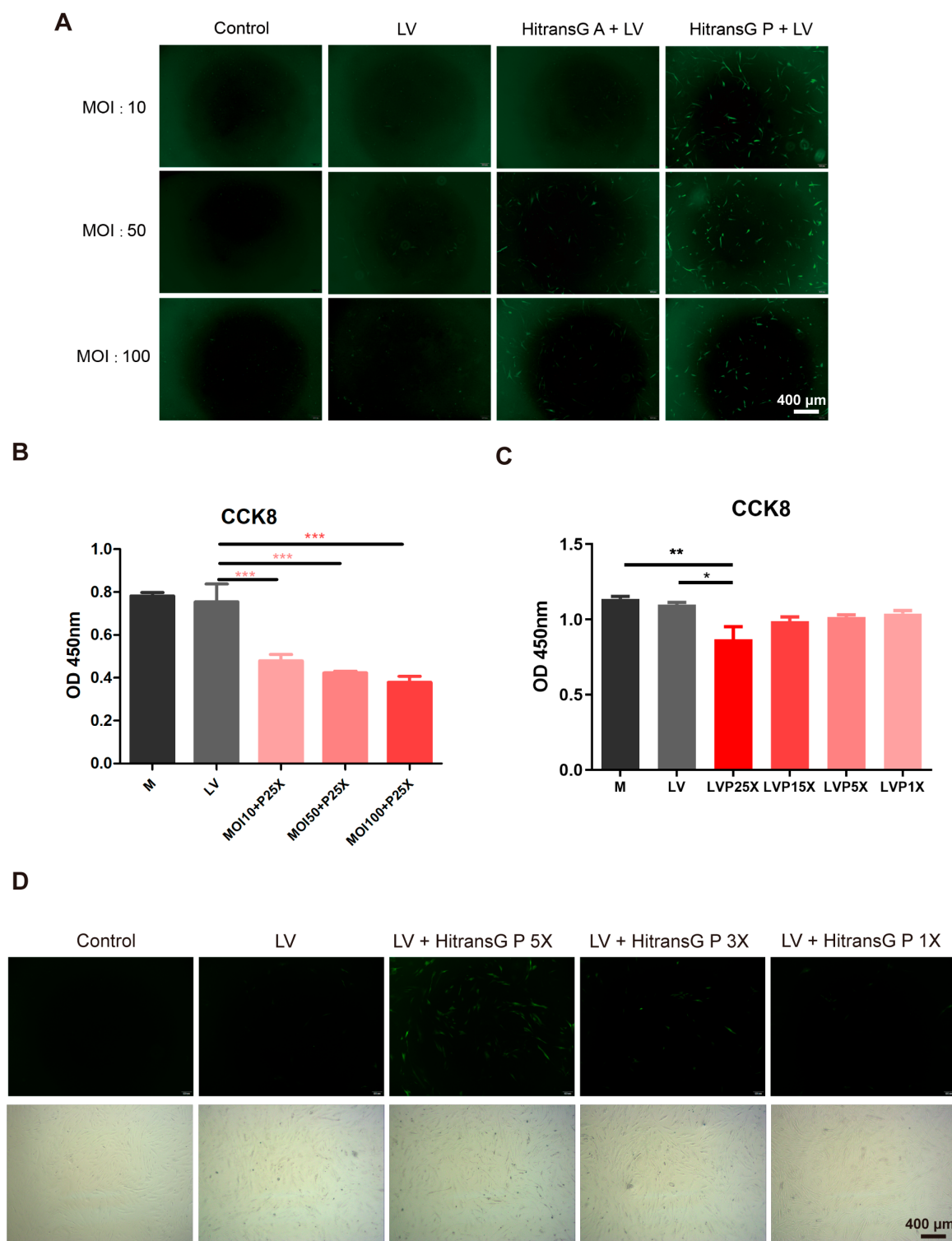

**Figure S1.** Lentiviral transfection of ADSCs.

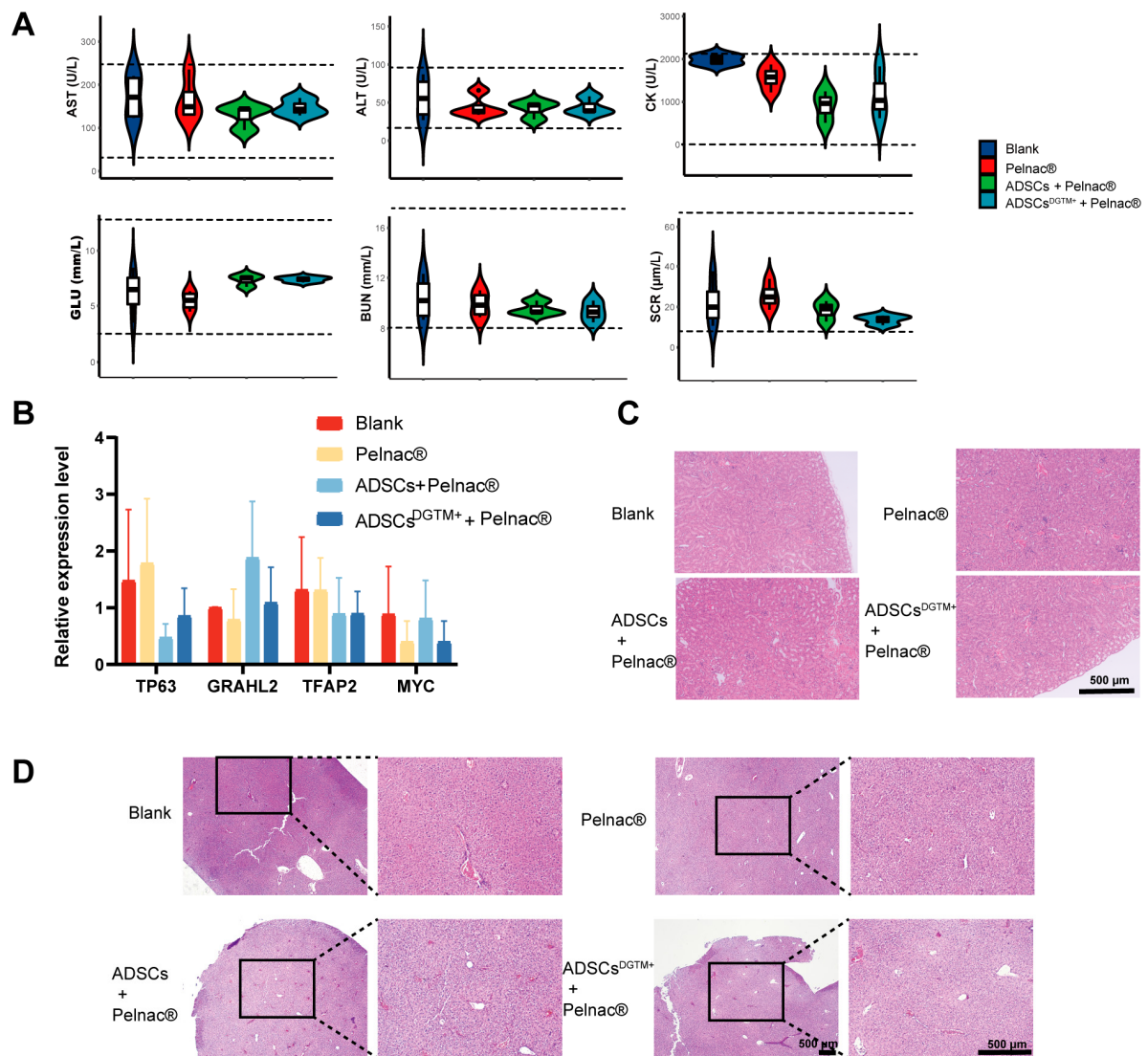

Figure S2. Safety evaluation.

Supplement: Supplementary file 1 [file biomedicines-12-02844-s001.zip › Supplementary Figures.pdf]
